# EPRS: Experience-Prioritized Reinforcement Scheduler in Edge Clusters

**DOI:** 10.3390/s26041168

**Published:** 2026-02-11

**Authors:** Shuya Tan, Tiancong Huang, Enguo Zhu, Jian Qin, Xiaoqi Fan

**Affiliations:** 1 School of Microelectronics and Communication Engineering, Chongqing University, Chongqing 400044, China; tanshuya@stu.cqu.edu.cn (S.T.); htc@cqu.edu.cn (T.H.); 202412131046t@stu.cqu.edu.cn (X.F.); 2 China Electric Power Research Institute, Beijing 100192, China; zhuenguo@epri.sgcc.com.cn

**Keywords:** load balancing, edge clustering, reinforcement learning, task scheduling

## Abstract

Edge computing has garnered significant attention in recent years due to its potential in distributed systems. However, the dynamic and heterogeneous nature of edge environments introduces substantial challenges for task scheduling. Conventional rule-based scheduling algorithms often fail to adapt to rapid load fluctuations, resulting in cluster load imbalance and suboptimal resource utilization. To address this issue, we propose a container-based edge cluster scheduling framework designed to enhance load balancing. Within this framework, we introduce an Experience-Prioritized Reinforcement Scheduler (EPRS), which leverages a priority-driven sample selection mechanism to facilitate focused learning of high-value samples. The EPRS dynamically monitors node resource states via a real-time resource monitor and optimizes multi-dimensional resource allocation by jointly considering node-level metrics (e.g., computational resources, memory pressure, storage performance, and container density) and task-specific resource requirements. To validate our approach, we implemented a system prototype integrated with the proposed framework and EPRS in a Kubernetes-based edge cluster. Experimental results demonstrate that the proposed method significantly improves multi-dimensional load balancing performance, achieving an average gain of 28.25% over existing reinforcement learning-based scheduling approaches and a 29.78% improvement compared with the traditional scheduling algorithm.

## 1. Introduction

Edge computing has emerged as a prominent research area in information technology due to its ability to address key limitations of traditional cloud computing. By decentralizing computation and bringing services closer to end-users, edge computing reduces bandwidth consumption and enhances local data security [1,2]. In this paradigm, edge cluster nodes deliver computing resources and application services within their proximity [3,4,5]. However, the inherently resource-constrained and dynamic nature of edge environments introduces significant challenges for efficient task scheduling. Poorly distributed workloads—such as concentrating tasks on a limited subset of nodes—can lead to cluster imbalance, resulting in node overload or degraded resource utilization. Consequently, optimizing load balancing in such constrained and volatile edge systems is critical for maintaining performance and stability.

Recent studies have adopted container technology to optimize resource management in resource-constrained edge computing environments [6,7]. Among various container solutions, Docker has emerged as the most widely-used platform, enabling lightweight, portable, and isolated application execution through containerization of binaries and their dependencies. For efficient container orchestration in edge clusters, Kubernetes has become the de facto framework for automated container deployment and management [8,9,10]. As an open-source, application-centric container orchestration system, Kubernetes provides extensive support for multiple container technologies, including Docker. However, with the continuous growth of application scale and business demands, critical challenges have emerged in container management, particularly regarding optimal resource allocation and load distribution. Effective scheduling mechanisms have consequently become essential for maintaining system performance and efficiency in expanding edge deployments.

Reinforcement learning, as a machine learning method that learns optimal policies by interacting with the environment, has shown strong application potential in several fields in recent years [11,12,13,14]. For the dynamic environment problem with random loads in edge cluster scenarios, reinforcement learning can utilize its learning ability for the overall environment [15,16,17]. By modeling and analyzing the state information of edge clusters and the request characteristics of workloads, it realizes the mining of the deep laws of cluster load balancing, which can provide intelligent decision support for the dynamic allocation and scheduling of resources.

In this study, we use Kubernetes edge clusters to simulate the task application and scheduling in the system under edge scenarios, and propose a novel EPRS scheduling algorithm for the challenge of load balancing multidimensional resources in dynamically changing environments in edge clusters. The task scheduling problem is tailored for diverse services, and real-time resource allocation is performed to improve the load balancing degree of the system, thus improving the stability and security of the system. The key contributions of this work include:

- We propose a four-dimensional dynamic resource sensing model for edge cluster scheduling, which jointly captures CPU utilization, memory pressure, disk I/O, and container density to characterize multidimensional resource states in Kubernetes-based edge environments.
- We design an Experience-Prioritized Reinforcement Scheduler (EPRS), a reinforcement learning–based scheduling framework with outcome-aware experience prioritization, in which the learning process emphasizes scheduling decisions that induce significant multidimensional resource imbalance or utilization variation, thereby improving adaptation to dynamic edge workloads.
- We implement the proposed EPRS in a real-world Kubernetes edge cluster and evaluate it under dynamic workloads, demonstrating its effectiveness in improving multidimensional resource utilization balance compared with baseline schedulers.

The remaining sections of this paper are organized as follows: Section 2 reviews related work on container-based edge computing frameworks and reinforcement learning-based scheduling approaches. Section 3 presents our proposed edge cluster framework in detail, including the architectural design of the Experience-Prioritized Reinforcement Scheduler (EPRS) and comprehensive system implementation details. In Section 4, we describe the experimental setup using real Kubernetes clusters, present our evaluation methodology, and analyze the results. Finally, Section 5 concludes the paper and outlines directions for future research.

## 2. Related Work

This section outlines the research background relevant to our work and reviews existing studies in two key areas: container-based edge computing frameworks and reinforcement learning-based scheduling algorithms.

### 2.1. Container-Based Edge Frameworks

In the past few years, many researchers have made studies on container-based edge computing architectures and Kubernetes is often used as a container orchestrator to play an important role in edge frameworks. Typically, the literature [18] implements a set of edge computing architectures based on container-based virtualization, integrating technologies from OpenStack, Kubernetes, Docker, and Ceph, and monitoring and evaluating cluster systems through the Ganglia Monitoring System. Ref.  [19] treated the virtual Kubelet as an executable platform or agent and proposed FLEDGE, a low-resource container orchestrator that demonstrated particularly lower memory requirements. The authors of [20], in order to advance federated learning based on Kubernetes, designed a framework in a cloud edge environment that isolates the network and enhances the security of the system. Ref. [21] proposed an enhanced load balancer with resource adaptive agents that makes load balancing decisions by monitoring the resource state of each Pod and the state of the worker nodes. Ref. [22] designed GAIKube container orchestrator that generates container scheduling, migration, dynamic vertical scaling, and switching of hosted application models that largely avoids server failures by balancing goals such as accuracy and cost.

While these studies have successfully developed Kubernetes-based edge computing frameworks, they primarily focus on architectural integration, orchestration mechanisms, and system-level optimization for edge or cloud–edge environments. In these frameworks, task scheduling is typically considered an operational component supporting the overall system design, with scheduling strategies often derived from heuristic policies or predefined resource indicators. Consequently, existing works emphasize platform-level efficiency and system coordination, rather than explicitly targeting the learning-driven optimization of multidimensional resource utilization under dynamic workloads and varying container density.

### 2.2. Reinforcement Learning-Based Container Scheduling

Reinforcement learning learns the optimal policy by interacting with the environment, and for clustered scenarios it can be utilized for its ability to capture the global system state. Typically, ref. [23] modeled the container placement problem as an objective optimization model or a graph-based network model to characterize container placement probabilities on edge servers, upon which multi-objective optimization or graph learning algorithms can be designed. Ref. [24] considered resource scalability and optimization in edge cluster systems and proposed an intelligent resource allocation strategy based on deep Q-networks (DQNs), which reduces average task service latency while maintaining fairness and efficiency of edge computing resources. The authors of [25] generalized single-task scheduling to multi-task scheduling through multi-agent reinforcement learning, alleviating the enlarged decision space and shortening average task completion time while improving resource efficiency in edge cloud systems. Ref. [26] designed EdgeOptimizer through a modular architecture as an edge cluster management platform, mainly targeting time-critical task scheduling using a dueling double deep Q network (D3QN) in edge–cloud collaborative scenarios. Ref. [27] formulated the Kubernetes scheduling problem as a Markov decision process and designed a DRS monitoring mechanism to sense node resource parameters, enabling a DQN-based scheduler to improve resource utilization and reduce load imbalance. Ref. [28] proposed an EEC system framework based on Kubernetes and Rancher, where a reinforcement learning-based scheduling algorithm minimizes task completion time and waiting time under high-load conditions. Ref. [29] presented a delay-aware reinforcement learning scheduling algorithm based on policy gradient and self-attention mechanisms for mobile edge computing, performing online container scheduling to minimize total task latency.

Beyond these studies, several recent works have incorporated prioritized experience replay into reinforcement learning-based scheduling frameworks. For example, ref. [30] applied prioritized experience replay to edge computing scenarios to optimize energy–latency trade-offs, while [31] demonstrated its effectiveness in cloud scheduling by accelerating convergence toward latency-aware policies. These studies confirm the benefits of experience prioritization for improving learning efficiency. However, their optimization objectives are primarily defined in terms of task-level performance metrics, such as execution delay or energy consumption, and the prioritization criteria are weakly coupled with the impact of scheduling decisions on system-wide resource load distribution.

Overall, existing reinforcement learning–based schedulers mainly focus on optimizing task-centric objectives, such as latency, energy efficiency, or average resource utilization. In contrast, the problem of multidimensional load balancing in edge cluster environments—especially under dynamic workloads and varying container density—has received comparatively less attention. In this work, we design an edge cluster scheduling framework that explicitly incorporates container density as part of the system state and integrates prioritized experience replay to emphasize scheduling experiences that significantly affect resource load distribution. By doing so, the proposed approach improves load balancing across edge nodes while avoiding single-resource overload. The proposed framework and algorithm are implemented and evaluated on an edge cluster testbed consisting of 11 physical nodes.

## 3. Model and Algorithm

**Figure 1:**
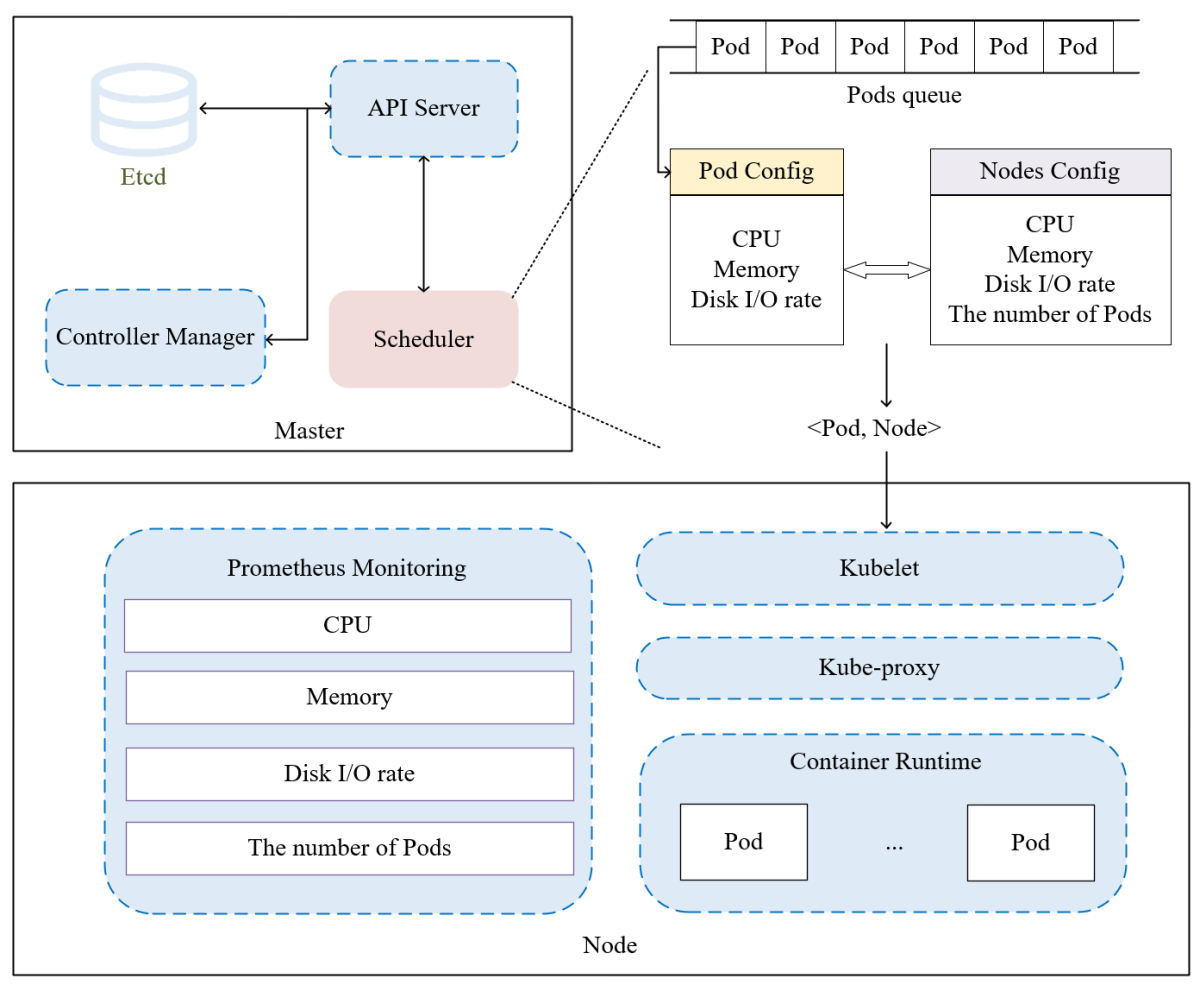
Scheduling flowchart.

In Kubernetes environments, tasks are encapsulated as containers within Pods—the smallest deployable computational units where co-located containers share resources and are scheduled collectively to worker nodes [32]. For our framework, we treat each Pod as an atomic task. The edge cluster architecture consists of a master node managing multiple worker nodes, with all task execution occurring on worker nodes. Figure 1 illustrates this fundamental workflow.

In this section, we first model the system for the scheduling problem in edge clusters, then introduce our proposed EPRS, and finally give a detailed description of our edge scheduling system architecture.

### 3.1. Modeling the Scheduling Problem in Edge Clustering

We construct edge clusters with one master node, *N* working nodes, and each node in the cluster has *M* real-time resource metrics, which are the node’s M−1 dimensional resources (e.g., CPU, memory, disk I/O rate) and the number of Pods running on the node. In our model, only the current utilization rates of the first M−1 resources are used in calculating the resource utilization, which reflects the current resource usage of the nodes. When calculating the degree of load imbalance in the system, the combination of the number of running Pods on a node avoids resource competition among multiple tasks on a single node, which can lead to a decrease in cluster resource utilization. We assume that there is at most one task to be deployed at the same moment *t*. The total resources of node n∈{1,2,…,N} can be denoted as ${ℜ}_{n}=\left\{re_{n}^{1},re_{n}^{2},\ldots ,re_{n}^{M-1}\right\}$, and its resource usage at moment *t* can be denoted as $U_{n}\left(t\right)=\left\{u_{n}^{1}\left(t\right),u_{n}^{2}\left(t\right),\ldots ,u_{n}^{M-1}\left(t\right)\right\}$. The resource demand of the Pod requested by the user at moment *t* can be denoted as $D\left(t\right)=\{d^{1}\left(t\right),d^{2}\left(t\right),\ldots ,d^{M-1}\left(t\right)\}$. The *m*-th dimension resource utilization $ru_{n}^{m}\left(t\right)$ on node *n* is denoted as:

(1) $$ru_{n}^{m}\left(t\right)=\frac{u_{n}^{m}\left(t\right)}{re_{n}^{m}}.$$

The average resource utilization of each node in the cluster at time *t* is defined as:

(2) $${\bar{u}}_{n}\left(t\right)=\frac{1}{M-1}\sum_{m=1}^{M-1}ru_{n}^{m}\left(t\right),\;n\in \left\{1,2,\ldots ,N\right\}.$$

The average resource utilization of the edge cluster system can be expressed as:

(3) $$\bar{u}\left(t\right)=\frac{1}{N}\sum_{n=1}^{N}{\bar{u}}_{n}\left(t\right).$$

To explicitly capture the impact of uneven task placement on cluster performance, resource utilization and Pod distribution are jointly considered in the scheduling model.

In addition to the average resource utilization, the load balancing of the cluster is another very important metric, and a load balanced cluster is more stable. The number of Pods on a node is also a very important metric for load balancing between nodes, denote the number of Pods running on node *n* at moment *t* as $p_{n}\left(t\right)$, then the total number of Pods running on the cluster at moment *t* can be denoted as $P\left(t\right)=\sum_{n=1}^{N}p_{n}\left(t\right)$, and the percentage of Pods running on node *n* can be denoted as:

(4) $$ru_{n}^{M}\left(t\right)=\frac{p_{n}\left(t\right)}{P(t)}$$

In calculating the degree of resource imbalance in the cluster, the standard deviation of the resource utilization between nodes with respect to the Pod share is used, denoted as:

(5) $$I\left(t\right)=\frac{1}{M}\sum_{m=1}^{M}\sqrt{\frac{1}{N-1}\sum_{n=1}^{N}{\left(ru_{n}^{m}\left(t\right)-\frac{1}{N}\sum_{n=1}^{N}ru_{n}^{m}\left(t\right)\right)}^{2}}$$

The metric I(t) measures the dispersion of multidimensional resource utilization across nodes, where a smaller value indicates a more uniform distribution. For node *n*, if the Pod requested at moment *t* is deployed to this node, then $b_{n}\left(t\right)=1$; otherwise $b_{n}\left(t\right)=0$. A task can only choose one node; the following needs to be satisfied:

(6) $$\sum_{n=1}^{N}b_{n}\left(t\right)=1$$

The resource utilization on the nodes in the cluster is updated after scheduling a task to a node:

(7) $$Lu_{n}^{m}\left(t\right)=\frac{u_{n}^{m}\left(t\right)+b_{n}\left(t\right)d^{m}\left(t\right)}{re_{n}^{m}}$$

When assigning Pods, upper and lower bounds are imposed on the post-scheduling resource utilization of each node to ensure stable and effective cluster operation. Specifically, the upper bound of 0.9 is introduced to prevent excessive resource contention among co-located Pods. In edge clusters with limited and tightly coupled resources, utilization levels approaching saturation may lead to CPU throttling, memory pressure, or I/O congestion, which can significantly degrade scheduling reliability and system responsiveness. Meanwhile, a lower utilization bound of 0.1 is applied to avoid persistently underutilized nodes caused by overly sparse task placement. Extremely low utilization levels indicate that the available resources of a node are insufficiently engaged in task execution, which may reduce the overall effectiveness of resource allocation at the cluster level. By constraining the post-scheduling utilization within a reasonable operating range, the scheduler is guided to balance workload distribution while maintaining stable multi-dimensional resource usage across nodes.

Based on the above system model and metric definitions, the task scheduling problem is formulated as an optimization problem that determines the target node for each arriving Pod. Our objective is to improve the overall performance of the edge cluster by maximizing the average resource utilization while minimizing the degree of load imbalance among nodes. Accordingly, the scheduling decision at time *t* is formulated as the following constrained optimization problem, which is subject to node-level resource capacity limits and task assignment feasibility constraints:

(8) $$\begin{array}{cccc} \max_{\left\{b_{n}\left(t\right)\right\}}\;\; & w_{1}\bar{u}\left(t\right)-w_{2}I\left(t\right)\\ s.t.\;\; & Lu_{n}^{m}\left(t\right)\geq 0.1,\; & \; & \forall n\in \{1,\ldots ,N\},\;\forall m\in \{1,\ldots ,M-1\},\\ \; & Lu_{n}^{m}\left(t\right)\leq 0.9,\; & \; & \forall n\in \{1,\ldots ,N\},\;\forall m\in \{1,\ldots ,M-1\},\\ \; & \sum_{n=1}^{N}b_{n}\left(t\right)=1,\\ \; & b_{n}\left(t\right)\in \left\{0,1\right\},\; & \; & \forall n\in \{1,\ldots ,N\}. \end{array}$$

Here, $b_{n}\left(t\right)$ is a binary decision variable indicating whether the Pod arriving at time *t* is assigned to node *n*. The coefficients $w_{1}$ and $w_{2}$ control the relative importance of average resource utilization and load imbalance, respectively.

The above formulation describes an ideal constrained optimization problem. In practice, directly enforcing hard constraints in reinforcement learning is non-trivial. Therefore, in the proposed EPRS, these constraints are incorporated into the reward function in the form of penalty terms, allowing the agent to learn feasible scheduling policies through interaction with the environment.

### 3.2. EPRS: Scheduling Algorithm Design

Deep reinforcement learning has received a lot of attention in solving complex scheduling problems; there are different deep reinforcement learning methods to accomplish task scheduling. Researchers [26,27] proved the effectiveness of DQN and D3QN for task scheduling in Kubernetes clusters, and we designed EPRS based on their research, aiming to achieve load-balancing in edge clusters for task scheduling. Task scheduling in edge clusters is a high-dimensional decision problem, where the optimal policy must consider dynamic resource availability, workload balancing, and long-term cluster stability. Traditional rule-based schedulers rely on static heuristics that may not be adapted to real-time resource fluctuations in the cluster. In this study, an online scheduling application is designed to solve the task scheduling problem in edge clusters by relying mainly on D3QN and Priority Experience Replay (PER).The system learns how to assign the to-be-scheduled Pods to the optimal nodes by observing the cluster resource status and Pod resource requirements in real-time. Using Prioritized Experience Replay (PER), the critical scheduling experience can be better learned to improve the load balancing of edge clusters while avoiding overloading of a single resource on a node.

In this paper, the edge cluster task scheduling problem is modeled as a Markov decision process: the state space *S*, the action space *A*, and the reward function *R*. At the moment *t*, the scheduling algorithm observes the environment state $s_{t}$ through the parameter acquisition module of the cluster and the resource demand of the task, selects the scheduling node $a_{t}$ in the action space according to the scheduling policy, obtains the reward $r_{t}\left(s_{t},a_{t}\right)$, and the environment state changes to $s_{t+1}$ after the scheduling module executes $a_{t}$, and then selects the next action according to the state $s_{t+1}$. The scheduling algorithm continuously interacts with the environment so that the model learns the ability of autonomous scheduling.

**Figure 2:**
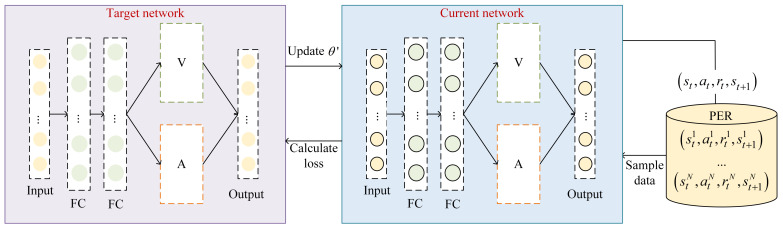
EPRS structure.

The structure of EPRS is shown in Figure 2.

The state space in the edge cluster task scheduling process, i.e., the state of the cluster node at the current moment and the amount of resources requested by the requested Pod, is obtained through the parameter collection module. The current state of node *n* can be represented as:

(9) $$U_{n}\left(t\right)=\left\{u_{n}^{1}\left(t\right),u_{n}^{2}\left(t\right),\ldots ,u_{n}^{M}\left(t\right)\right\}$$

where the first M−1 elements represent the current resource utilization of the node, where the resource metrics include CPU, memory, and disk I/O rate, respectively, and the *M*-th element represents the number of Pods currently running on the node.

The state of the Pod to be scheduled at time *t* is denoted as:

(10) $$D\left(t\right)=\left\{d^{1}\left(t\right),d^{2}\left(t\right),\ldots ,d^{M}\left(t\right)\right\}$$

where the first M−1 elements represent the amount of requests for different resources by Pods, where the resource metrics include CPU, memory, and disk I/O rates, respectively, and the *M*-th element has a value of 1, and the number of Pods on the corresponding node will be added by one after this Pod is scheduled.

The state space $s_{t}$ of a cluster is a one-dimensional vector, denoted as:

(11) $$s_{t}=\left[U_{1}\left(t\right),U_{2}\left(t\right),\ldots ,U_{N}\left(t\right),D\left(t\right)\right]$$

During the task scheduling process, the scheduling algorithm acts as an intelligent body and takes action every time it receives a new scheduling request, in our system, the action represents that the current task will be scheduled on a particular worker node, so the action space is all the worker nodes on the cluster.

(12) $$a_{t}\in \left\{node_{n}\right\},n\in \left\{1,2,\ldots ,N\right\}$$

After selecting an action, the reward function $r_{t}\left(s_{t},a_{t}\right)$ is calculated based on the action node and the state space. To optimize cluster performance, the system seeks to maximize average resource utilization and reduce load imbalance. A weighted sum of these two metrics is employed to determine the reward. Since when allocating Pods, we hope that after allocating Pods to specific nodes, the individual resource utilization of the nodes will not exceed 0.9 to avoid resource competition, when the individual resource utilization of a node in the cluster exceeds 0.9, a penalty factor penalty1 is introduced. We want the resource utilization of a single node to be not less than 0.1 after assigning a Pod to avoid resource wastage, and when the individual resource utilization of a node in the cluster is less than 0.1, a penalty factor penalty2 is introduced. The final reward function is defined as:

(13) $$r_{t}=w_{1}\bar{u}\left(t\right)-w_{2}I\left(t\right)-\mathrm{penalty}1-\mathrm{penalty}2$$

where $w_{1}$ and $w_{2}$ are the coefficients.

The penalty terms correspond to soft constraint violations and are defined as follows.

(14) $${\mathrm{penalty}}_{1}=\left\{\begin{array}{cc} 0, & \mathrm{if}\;\forall n,m:Lu_{n}^{m}\left(t\right)\leq 0.9\\ c_{1}, & \mathrm{otherwise} \end{array}\right.$$

(15) $${\mathrm{penalty}}_{2}=\left\{\begin{array}{cc} 0, & \mathrm{if}\;\forall n,m:Lu_{n}^{m}\left(t\right)\geq 0.1\\ c_{2}, & \mathrm{otherwise} \end{array}\right.$$

Here, $c_{1}$ and $c_{2}$ are fixed penalty constants that determine the severity of utilization constraint violations. This formulation transforms the hard utilization constraints in Equation (8) into soft penalties, enabling stable policy learning in a reinforcement learning setting.

D3QN combines the ideas of Dueling Network and Double Q-Learning, which enables the neural network to better learn the value of the state and the relative value between different actions, and improves the stability of the algorithm’s performance. D3QN belongs to the variant of the classical DRL algorithm DQN based on value estimation.

The D3QN consists of a current network and a target network, which are Q-networks with different parameters but the same structure, the evaluation network with parameter θ and the target network with parameter $\theta^{\prime }$. The D3QN reconstructs the Q-value function, denoted by decoupling the state values and the action advantages, at the moment *t*:

(16) $$Q_{t}\left(s_{t},a_{t};\theta ,p,q\right)=V_{t}\left(s_{t},a_{t};\theta ,q\right)+\left(A\left(s_{t},a_{t};\theta ,p\right)-\frac{1}{N_{A}}A\left(s_{t},a_{t}^{*};\theta ,p\right)\right)$$

where $V_{t}\left(\cdot \right)$ is the value function, A(·) is the action dominance function, $a_{t}^{*}$ is all actions that can be selected at moment *t*, and $N_{A}$ is the number of actions. *p* and *q* are the network parameters of the value function and action dominance function, respectively.

The target value is calculated as:

(17) $$y_{t}=r_{t+1}+\gamma Q\left(s_{t+1},\arg\max_{a}Q\left(s_{t+1},a;\theta \right);\theta^{\prime }\right)$$

The evaluation network is utilized to obtain the action corresponding to the optimal action value in the state $s_{t+1}$, and then the target network is utilized to calculate the action value of the action to obtain the target value.

In order to make the intelligences acquire key experiences more efficiently and quickly, it is crucial to improve the sampling efficiency and data utilization. Experience pooling is a key technique in which at each time step *t*, the model first puts its past experience (S,A,R) into the experience pool and then randomly samples a fixed number of samples from this experience pool for Q function updating. The experience pool can reuse the experience of each time step to learn the Q-function, which improves the efficiency of sample utilization, avoids the samples used for network updating being derived solely from the previous strategy, and can break the correlation between the data, which can reduce the variance during the learning process to improve the stability of learning. In traditional experience pooling a random sampling method is usually used to select samples, which assumes that each sample in the pool contributes equally to model training. However, in reality, the data in the pool have different impacts on the effectiveness of training. At the beginning of the scheduling task, the experience pool may contain a large number of bad attempts and a small number of optimal schedules. If uniform sampling is used, the chances of drawing valuable successful samples will be small, making the training progress slow. A smarter sampling strategy is therefore needed to ensure that the training process makes more efficient use of those samples that are most helpful in improving performance.

Prioritized Experience Replay (PER) is a technique that prioritizes experience samples in the experience pool. This technique allows important state transfer experiences to be used more frequently for updating current strategies can optimize sample efficiency and accelerate the learning of key strategies. The core idea is to consider the importance of different state transfer data through the time difference (TD) error δ. The TD error is the difference between the expected future reward and the value of the current action value function, denoted as:

(18) $$\delta_{t}=r_{t+1}+\gamma \max_{a}Q\left(s_{t+1},a_{t+1}\right)-Q\left(s_{t},a_{t}\right)$$

The priority of the *j*-th experience at decision moment *t* is defined as follows:

(19) $$P_{TD}^{j}=\left|\delta_{j}\right|+\varepsilon_{td}$$

where $\varepsilon_{td}$ is a small positive number that prevents the TD error of the experience selected during training from being 0, resulting in not being valued by the Q-strategy. $\delta_{j}$ is the TD error of the *j*-th experience transitioning from state $s_{t}$ to the next state $s_{t+1}$.

The probability that empirical *j* is drawn based on TD error prioritization is defined as:

(20) $$P_{TD}\left(j\right)=\frac{P_{TD}^{j}\alpha_{td}}{\sum_{i=1}^{N_{s}}P_{TD}^{i}\alpha_{td}}$$

where $N_{s}$ is the capacity of the experience pool. $\alpha_{td}$ is the priority index, which reflects how much the priority $P_{TD}^{j}$ of a learned experience affects the probability of that experience being selected, when $\alpha_{td}=0$ means sampling all experiences uniformly, and $\alpha_{td}$ is close to 1 means focusing on priority-based sampling.

The loss equation can be expressed as:

(21) $$L\left(\theta \right)=\frac{1}{N_{s}}\sum_{j=1}^{N_{s}}\delta_{j}^{2}\eta_{j}$$

where:

(22) $$\eta_{j}={\left(\frac{1}{N_{s}}\cdot \frac{1}{P_{TD}\left(j\right)}\right)}^{\beta }$$

β∈[0,1] represents the degree of correction. In the conventional experience replay mechanism, the replayability of different experiences is not considered, i.e., each experience has the same chance to update the weights of the neural network. In contrast, in prioritized experience playback, different priorities have different weights, and different update weights are calculated for different experiences by introducing coefficients. The algorithm in this paper needs to add priority weights to the samples and uses SumTree to store the samples.

The single-step time complexity of the traditional D3QN algorithm is O(B·L·D), where *B* is the batch size, *L* is the number of network layers, and *D* is the number of neurons per layer. The time complexity of the algorithm proposed in this paper is $O(B\cdot L\cdot D+\log N_{s})$, where the additional $\log N_{s}$ term is introduced by prioritized experience replay due to the use of the SumTree structure. Although the state representation aggregates information from all nodes, the complexity analysis focuses on the number of network inference steps per decision, while the impact of state dimensionality is reflected in the fixed network width under the experimental settings.

In our implementation, the replay buffer size $N_{s}$ is bounded by 1000, making the $\log N_{s}$ sampling overhead negligible compared with the network computation cost.

### 3.3. Edge Cluster System Design

**Figure 3:**
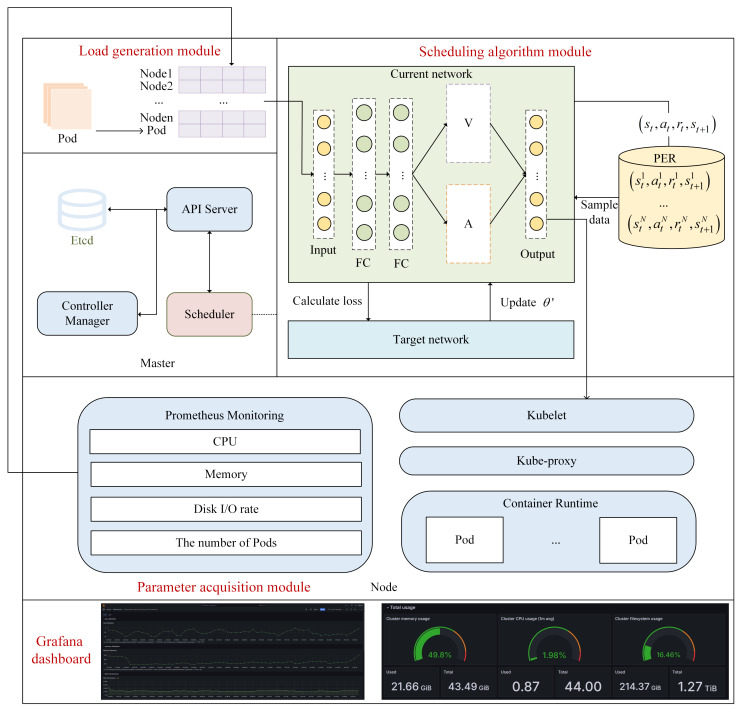
Overall system architecture.

The architecture of the edge scheduling system consists of a load generation module, a parameter collection module, a scheduling algorithm module, and a request scheduling module, which are non-intrusive and rely on add-ons and API interfaces that do not disrupt the native Kubernetes architecture. The overall system framework designed is shown in Figure 3.

Load Generation Module: Shell scripts are employed to generate Pod tasks, and stress-ng is utilized to emulate realistic resource pressure induced by workloads. The generated tasks consume corresponding types of node resources, thereby affecting the real-time resource status of the nodes. This module enables users to configure heterogeneous workload requests according to different resource requirements.

Parameter Collection Module: Node-exporter is deployed to collect hardware- and operating-system-level metrics of cluster nodes, including CPU utilization, memory usage, and disk I/O statistics. Kube-state-metrics is adopted to obtain Pod-related information, such as the number of running Pods and their execution states. Prometheus is deployed within the cluster to aggregate monitoring data, enabling real-time access to resource utilization and Pod statistics through query interfaces exposed to the service orchestration module. Grafana is integrated for visualizing the collected metrics, facilitating the observation and comparison of node-level resource utilization under different scheduling algorithms.

Scheduling Algorithm Module: The Kubernetes client, implemented in Python 3.10, is initialized via the config.load_kube_config() interface to establish communication with the cluster. Pods in the Pending state are continuously monitored using the watch.Watch() mechanism. For each scheduling request, the module retrieves the current cluster resource state, including node-level utilization metrics and the number of active Pods, as well as the resource requirements specified by the incoming Pod. The aggregated state information is then fed into the proposed EPRS scheduling algorithm, which determines the scheduling action by selecting an appropriate worker node. The selected node is subsequently passed to the request scheduling module to complete the scheduling decision.

Request Scheduling Module: After the scheduling algorithm coordinates the node with the current task, it passes the selection result into the Request Scheduling Module, which connects to the k8s cluster master node by using the config.load_kube_config() method and places the task to run on the selected node.

## 4. Tests and Performance Evaluation

### 4.1. System Setup

**Figure 4:**
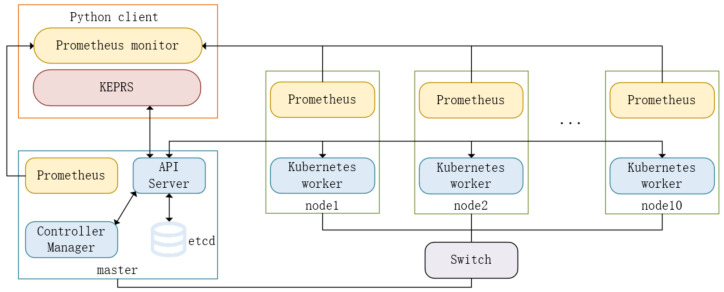
Design of the test platform.

**Figure 5:**
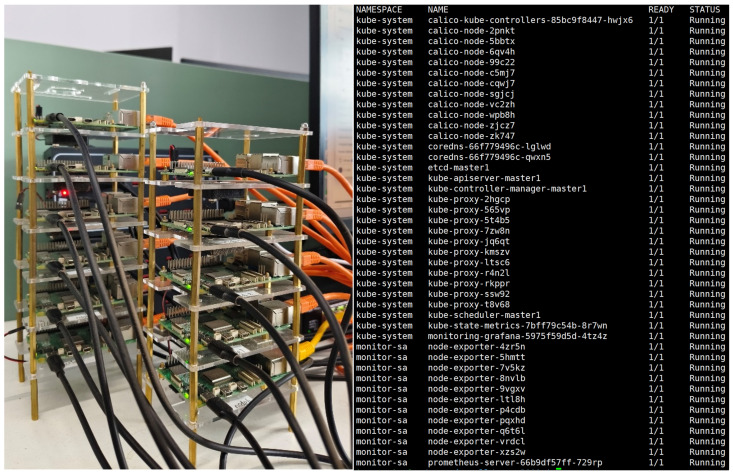
Physical node and related components on the node.

Our edge clustering system consists of 11 Raspberry Pi 5, 1 network switch and 1 PC host. One Raspberry Pi 5 is the master node and 10 Raspberry Pi 5s are working nodes. The network switch is a Riptide RG-ES116G-E gigabit switch with 16 ports. The nodes share the network and connect to each other through the switch. A host computer acts as a Python 3.10 client which is used to implement the scheduling algorithm. Each Raspberry Pi 5 is configured with a quad-core ARM Cortex-A76 @ 2.4 GHz, a memory size of 4 GB, a disk capacity of 128 GB, and a maximum disk I/O rate of about 30 MB/s. The Raspberry Pi uses the Debian operating system, and a Kubernetes cluster is deployed on the nodes. The design of the testbed is shown in Figure 4. The physical node and related components on the node are shown in Figure 5.

### 4.2. Hyperparameter Sensitivity Analysis

We first investigate the settings of various parameters in EPRS, including the designs for the Alpha, the exploration rate, and the learning rate (LR). We observe the changes in the reward values of the model for different parameter designs.

**Figure 6:**
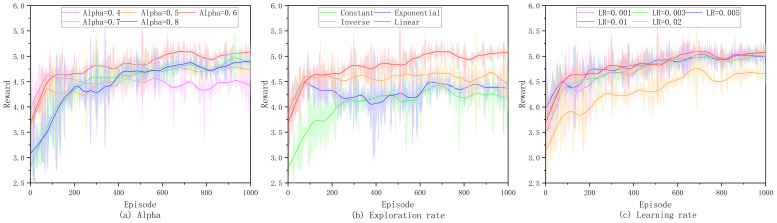
Sensitivity analysis of EPRS under different hyperparameter settings: (**a**) prioritization index α; (**b**) exploration rate decay strategies; (**c**) learning rate.

The impact of the prioritization index Alpha is illustrated in Figure 6a. At Alpha of 0.4 and 0.5, the algorithm tends to sample more uniformly and converges faster initially, but it results in an under-optimized strategy due to weakening the sampling opportunities of key samples. At Alpha of 0.7 and 0.8, the algorithm converges slowly initially. We finally choose a priority index of 0.6 to ensure the stability and convergence speed of the algorithm.

For the test of exploration rate, we try four different test schemes, and the performance of the model under fixed exploration rate (Constant), exponential decay of exploration rate (exponential), inverse time decay of exploration rate (inverse), and linear decay of exploration rate (linear) are compared in Figure 6b. The fixed exploration rate (Constant) leads to insufficient exploration in the early stage, and the model falls into local optimal solutions and fails to converge to the optimal policy. When the exploration rate decays exponentially and inverse time, the exploration rate decreases faster at the beginning, leading to insufficient exploration in the early stage. So we choose the linear decay method and set the exploration rate to decrease linearly from 0.9 to 0.1.

When the learning rate is set to 0.02, the stability of the model is not good enough. When the learning rate is less than 0.01, the convergence speed is slower compared to 0.01. The effect of different learning rates on model convergence is shown in Figure 6c. We set the learning rate to 0.01 to both converge quickly and avoid violent oscillations.

We set the degree of correction for prioritizing experience playback in the algorithm to 0.4 and $\varepsilon_{td}$ to $1\times 10^{-6}$ to avoid zero priority. To make the reward function easy to observe, we set $w_{1}$ and $w_{2}$ to 10. The penalty constants $c_{1}$ and $c_{2}$ are configured to reflect the asymmetric impact of different utilization violations. Resource over-utilization may lead to severe contention and instability at the node level, whereas under-utilization mainly results in reduced efficiency. Therefore, $c_{1}$ is set larger than $c_{2}$ to impose a stronger penalty on exceeding the upper utilization threshold. In our experiments, $c_{1}$ is set to 0.5 and $c_{2}$ is set to 0.25.

### 4.3. Experiments

We use the designed EPRS to schedule the requested tasks and compare it with the default Kubernetes scheduler, DQN [27], Dueling DQN, Double DQN, and D3QN [26]. We first train the simulation with random workloads and then verify the effectiveness of our proposed algorithm by testing the trained model under different workloads.

**Figure 7:**
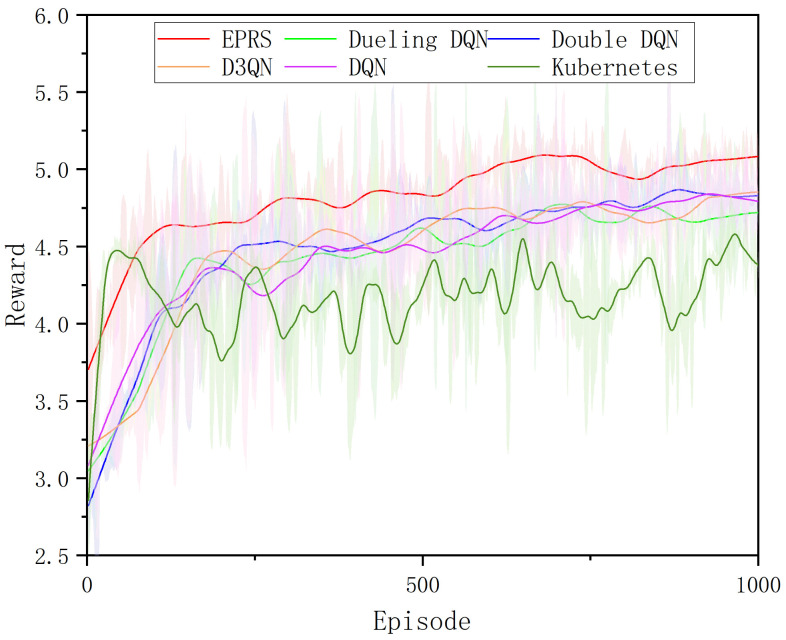
Rewards for different algorithm training.

We first train the EPRS model by generating random loads because the resource randomness of the loads can simulate more diverse cluster situations and make the model more robust in real and complex environments. During training, each Pod has a random combination of CPU applications of 100 milli-cores, 200 milli-cores, or 300 milli-cores; memory applications of 64 Mi, 128 Mi, or 256 Mi; and disk I/O rates of 1 MB/, 2 MB/s, or 3 MB/s. To ensure the reproducibility of the experiments, a random seed was set to ensure that all tasks trained by each algorithm remained consistent. One task is applied every 15 s, and the duration of each task is 4 min, and 1000 rounds are trained. The results are shown in Figure 7.

Figure 7 shows the variation of reward values for 1000 rounds of EPRS training, and it can be observed that the training process of EPRS is more successful. The reward values of EPRS are improved by 8.44% on average compared to D3QN and 8.51% compared to DQN. Due to the introduction of prioritized experience playback in EPRS, it can learn the key scheduling experience better and its convergence speed is faster.

The models we trained were put under different workloads to test the effectiveness of the models for system load balancing. The following three sets of workloads were constructed:

Group I: Workloads with the same amount of resource requests at the same time interval. In this case, a Pod is generated every 15 s, the amount of CPU resources requested by the Pod is 300 milli-cores, the memory is 256 Mi, and the disk I/O rate is 3 MB/s. The duration of each Pod is 4 min, and a total of 100 Pods are tested. A total of 100 Pods are tested to simulate the processing of the same task periodically under a stable load by this set of workloads.

Group II: Workload with random resource requests at the same time interval. In this case, a Pod is generated every 15 s, and each Pod has a CPU request of 100 milli-cores, 200 milli-cores, or 300 milli-cores, a memory request of 64 Mi, 128 Mi, or 256 Mi, and a disk I/O rate of 1 MB/s, 2 MB/s, or 3 MB/s, with resource requests independently sampled from predefined discrete candidate sets following a uniform distribution. Random seeds are set when generating tasks to ensure that all tasks trained by each algorithm remain consistent. Each Pod lasts for 4 min, and a total of 100 Pods are tested. This set of workloads simulates periodic processing of different tasks and evaluates the efficiency of scheduling algorithms under diverse task resource demands.

Group III: Workloads with random resource requests that conform to a Poisson distribution. The task arrival intervals are generated through an exponential distribution to realize a Poisson process with parameter λ=0.0667, corresponding to an average interval of 15 s thus constructing a chi-square Poisson process. The number of task arrivals obeys the Poisson distribution Poisson(λt), simulating the arrival pattern of random events in the real world. Each Pod has a CPU request of 100 milli-cores, 200 milli-cores or 300 milli-cores; a memory request of 64 Mi, 128 Mi or 256 Mi; a disk I/O rate of 1 MB/, 2 MB/s or 3 MB/s; and a randomly generated resource request. Random seeds are set when generating task resources to ensure that all tasks trained by each algorithm remain consistent. Random seeds are also used when generating task time intervals to ensure consistent time intervals between workloads tested by different algorithms. The duration of each Pod is 4 min, and a total of 100 Pods are tested. According to the mathematical model in queuing theory, the use of Poisson distribution to simulate the workloads can truly reflect the arrival pattern of random events in the real world. The Poisson distribution describes the probability of an independent event occurring at a fixed time interval, and its memoryless and sparse properties make it well suited for simulating bursty tasks such as network requests, user accesses, or micro-service invocations. By setting the Poisson process with an average interval of 15 s, workloads with randomized task arrival times but controllable overall loads can be generated to test the performance of scheduling algorithms under unpredictable stress.

In calculating the imbalance, we use the standard deviation of the utilization of a certain resource on different nodes in the cluster as the load imbalance, and to make the result easy to observe, we multiply the standard deviation by 100 to represent it. Since the utilization in the above graph will be smoother in the middle section, we take the node resource utilization in the middle smooth twenty minutes to calculate the load imbalance. The node resource utilization is obtained every 30 s and the load imbalance is expressed by calculating 100 times the standard deviation of the three resources under different algorithms.

#### 4.3.1. First Set of Experiments

**Figure 8:**
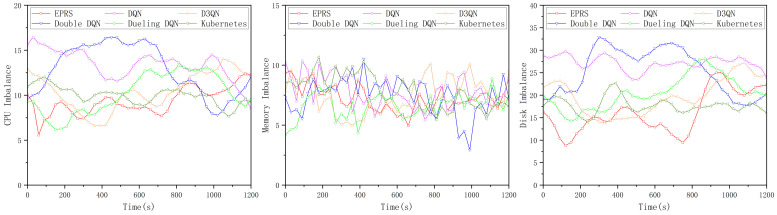
Load imbalance of the three resources in the first set of experiments.

In the first set of experiments, the load imbalance of the three resources (CPU, memory, and disk I/O rate) is shown in Figure 8.

**Figure 9:**
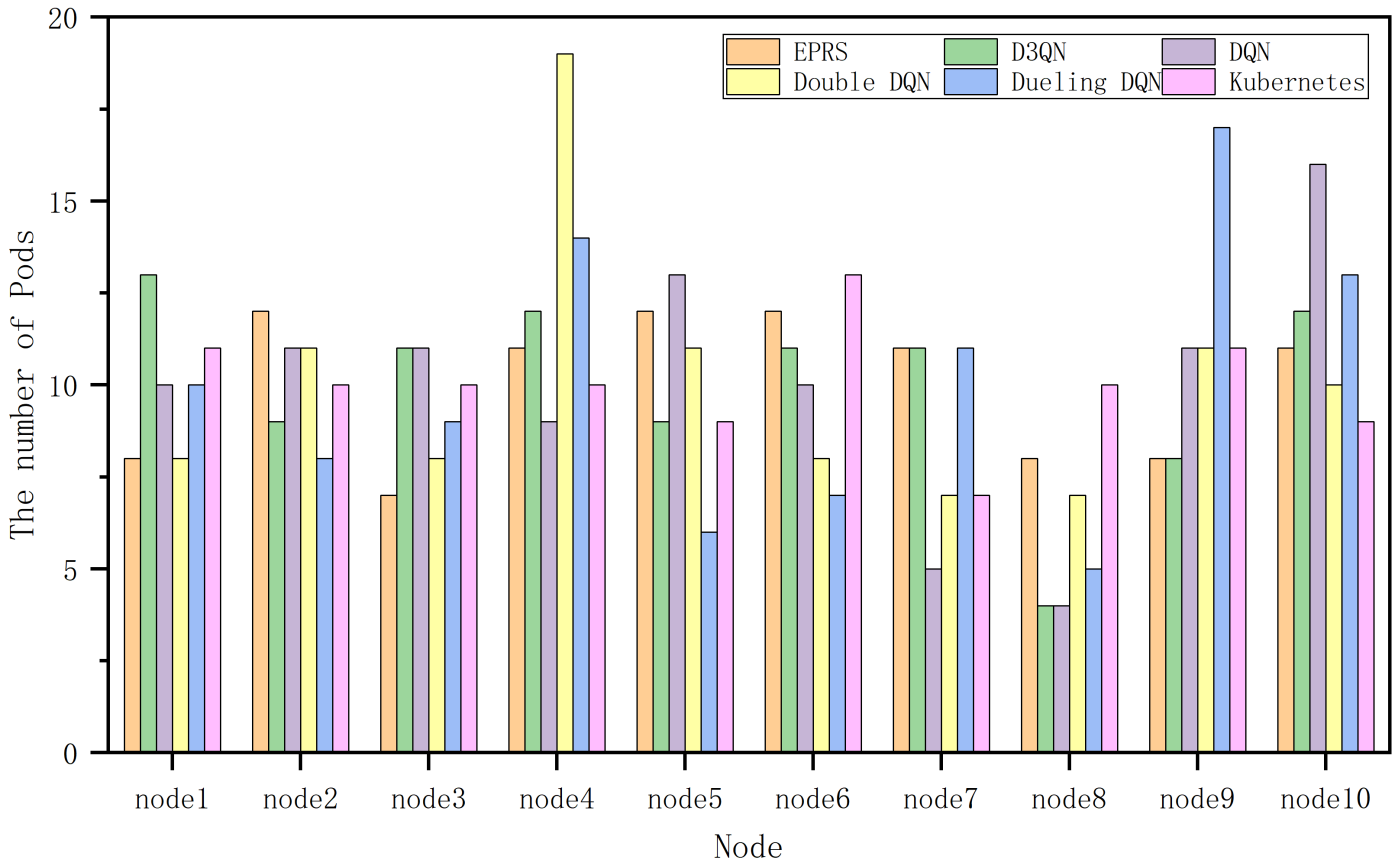
Number of Pods on each node in the first set of experiments.

In Figure 8, EPRS performs well in terms of CPU resources and disk I/O resources, and the gap with other algorithms in terms of memory resources is not significant, and the distribution of 100 Pods on nodes in the first group is shown in Figure 9.

**Figure 10:**
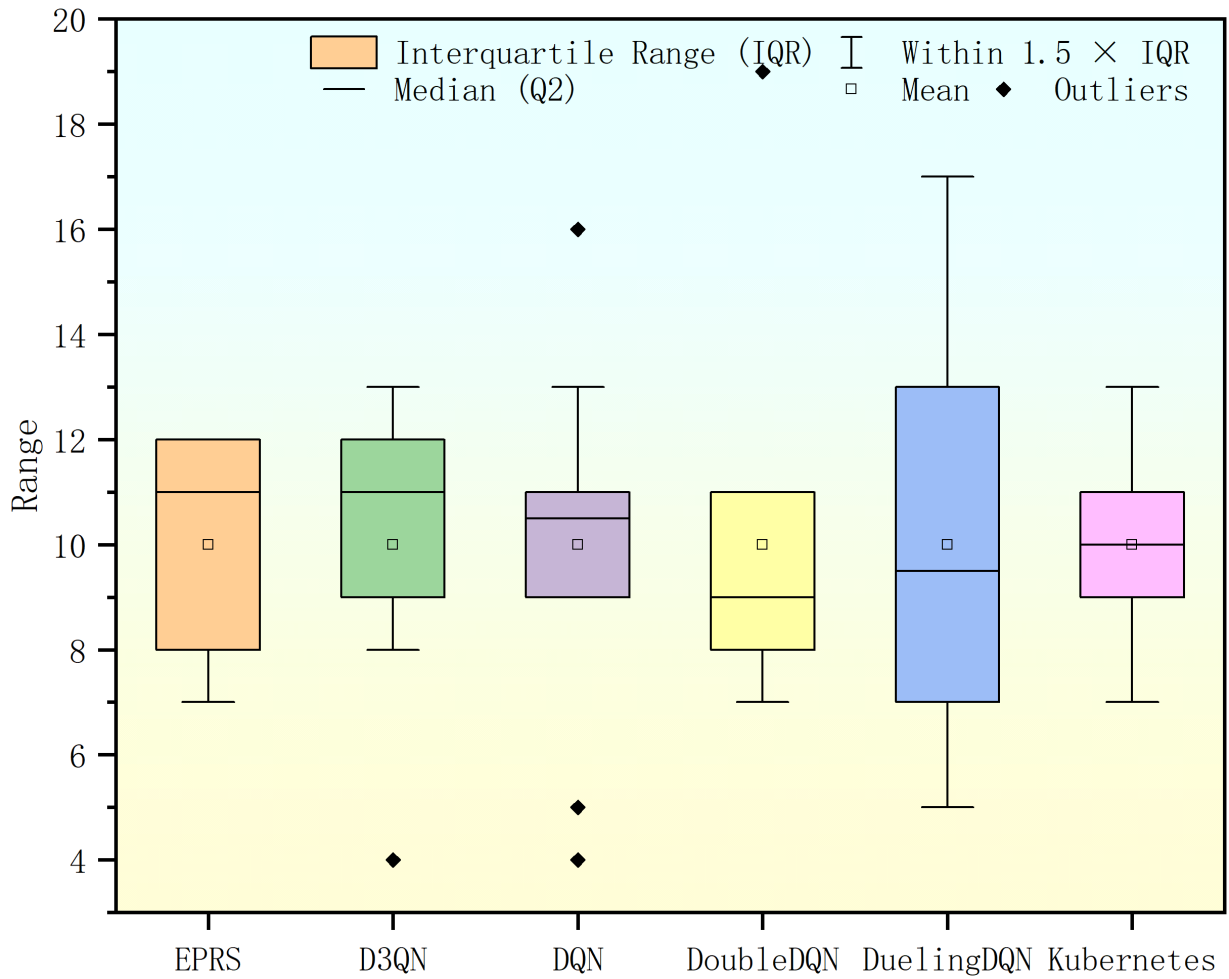
Range of Pod distribution in the first set of experiments.

Under this set of workloads, EPRS with D3QN performs better compared to other algorithms with outliers in DQN and excessive outliers in Double DQN, as shown in Figure 10.

#### 4.3.2. Second Set of Experiments

**Figure 11:**
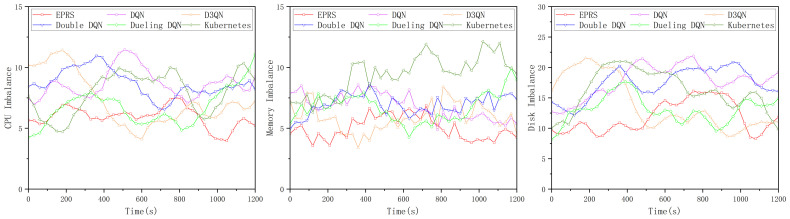
Load imbalance of the three resources in the second set of experiments.

In the second set of experiments, the load imbalance of the three resources (CPU, memory, and disk I/O rate) is shown in Figure 11.

**Figure 12:**
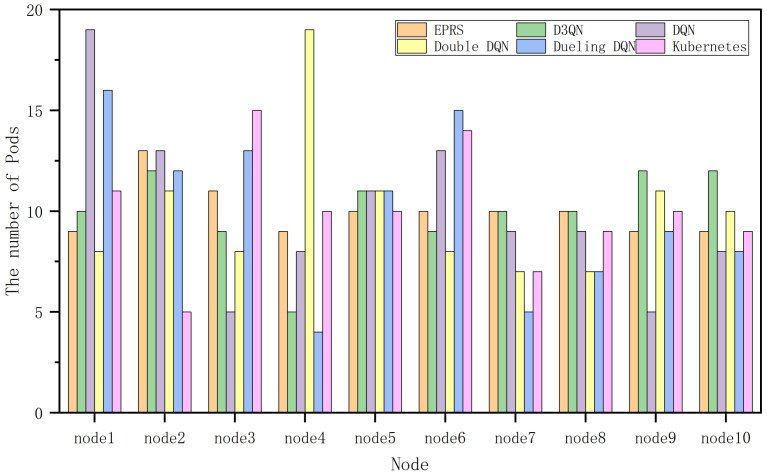
Number of Pods on each node in the second set of experiments.

In Figure 11, EPRS performs better in all the three resources and the distribution of 100 Pods on nodes in the second group is shown in Figure 12.

**Figure 13:**
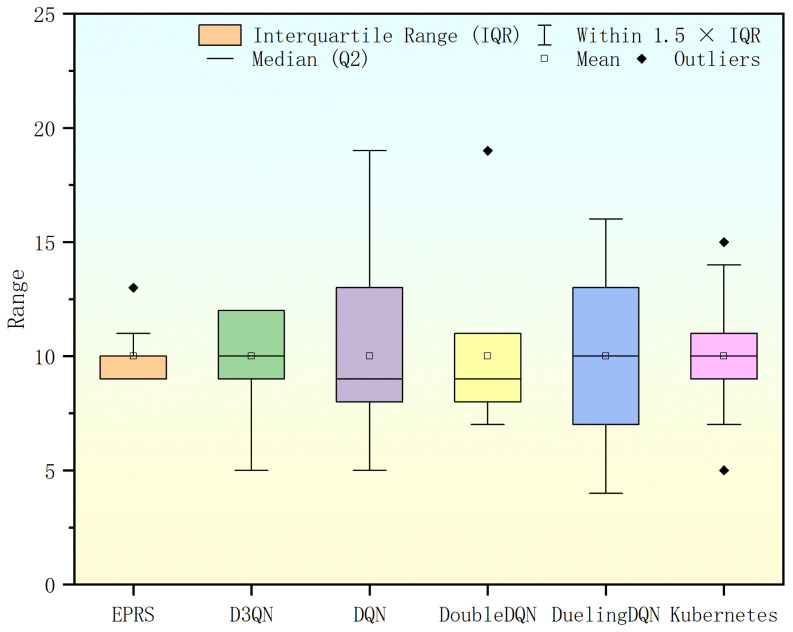
Range of Pod distribution in the second set of experiments.

In this set of workloads, EPRS performs best in terms of task distribution equalization, as shown in Figure 13.

#### 4.3.3. Third Group of Experiments

**Figure 14:**
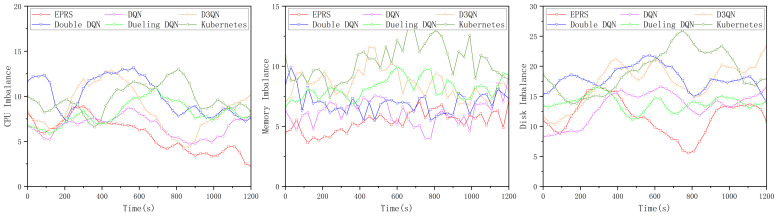
Load imbalance of the three resources in the third set of experiments.

In the third set of experiments, the load imbalance of the three resources (CPU, memory, and disk I/O rate) is shown in Figure 14.

**Figure 15:**
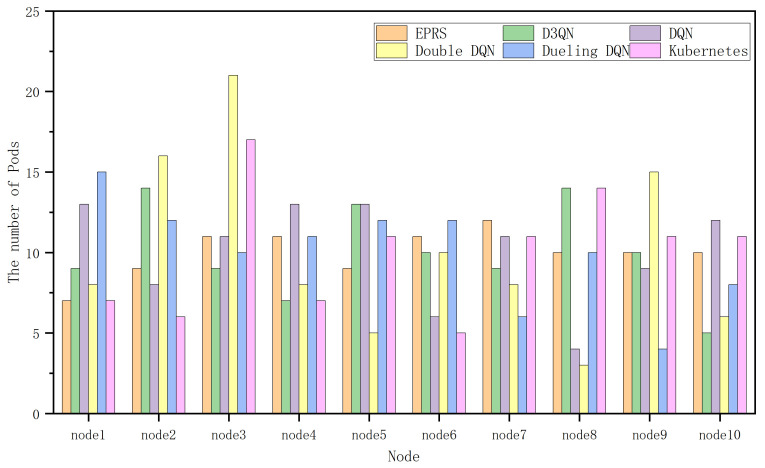
Number of Pods on each node in the third group of experiments.

In Figure 14, EPRS performs better in all the three resources and the distribution of 100 Pods on nodes in the third group is shown in Figure 15.

**Figure 16:**
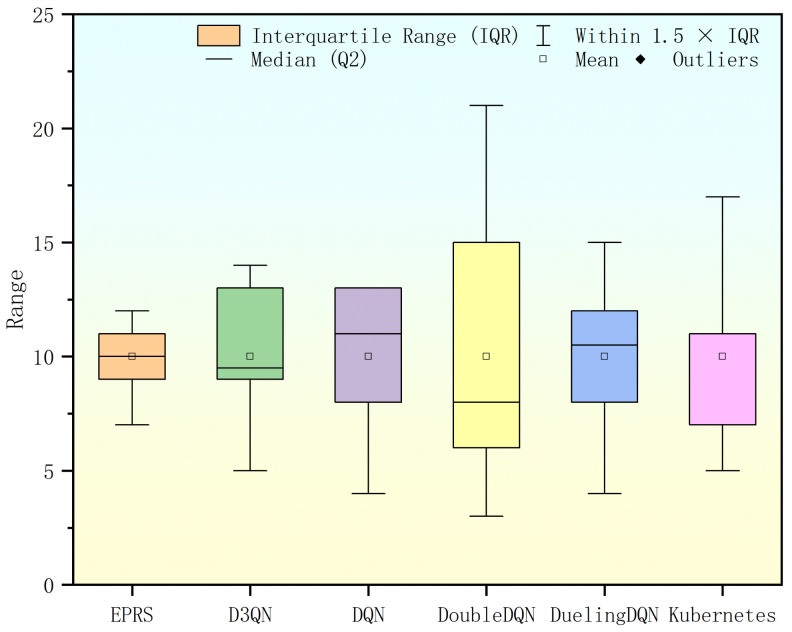
Range of Pod distribution in the third set of experiments.

Under this set of workloads, EPRS performs more balanced in terms of task distribution, as shown in Figure 16.

#### 4.3.4. Performance Analysis of Scheduling Algorithms

To quantitatively evaluate the load balancing performance of different scheduling algorithms, the load imbalance is measured as 100 times the standard deviation of the utilization ratios of CPU, memory, disk I/O resources, as well as the distribution of Pods across nodes. Table 1 reports the average load imbalance of different algorithms over a 20 min scheduling period under three workload groups.

From the experimental results under three workload groups, EPRS consistently achieves superior load balancing performance across most resource dimensions. In the first set of experiments, EPRS reduces the load imbalance of CPU, memory, disk I/O resources, and the number of Pods by 11.16%, 2.69%, 18.29%, and 23.81%, respectively, compared to D3QN, with an average improvement of 13.99%. Compared to DQN, the corresponding improvements reach 30.43%, 7.62%, 40.66%, and 42.79%, with an average improvement of 30.38%. EPRS achieves better load balancing performance than the other algorithms in all dimensions, except that its memory imbalance is higher than that of Dueling DQN and its performance on Pod density is inferior to Kubernetes.

In the second set of experiments, EPRS achieves the best load balancing performance in all dimensions. Compared with D3QN, the load imbalance of CPU, memory, disk I/O, and Pods is reduced by 19.01%, 14.76%, 15.40%, and 40.84%, respectively, with an average improvement of 22.5%. Compared with DQN, the corresponding improvements reach 31.56%, 26.31%, 33.17%, and 70.42%, with an average improvement of 40.37%.

In the third set of experiments, EPRS again outperforms the baseline algorithms across all dimensions. Compared to D3QN, EPRS improves the load imbalance of CPU, memory, disk I/O resources, and the number of Pods by 33.97%, 36.88%, 32.93%, and 51.97%, respectively, with an average improvement of 38.94%. Compared to DQN, the corresponding improvements are 14.20%, 13.04%, 12.89%, and 55.28%, with an average improvement of 23.85%.

**Figure 17:**
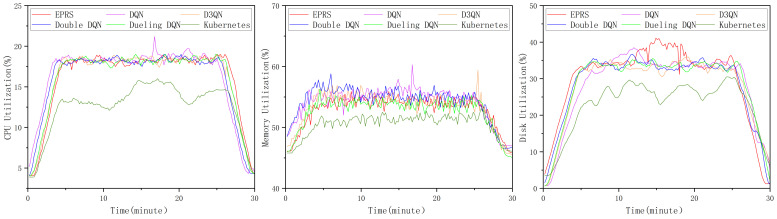
Average utilization of the three resources in the first set of experiments.

Figure 17 shows the average utilization of the three resources on all nodes for the first set of different algorithms.

The experimental results show that all reinforcement learning–based schedulers converge to comparable levels of average resource utilization. None of the learning-based methods causes persistent resource overloading on individual nodes, and thus no significant degradation of overall cluster utilization is observed. Consequently, the final average utilization achieved by different reinforcement learning algorithms remains largely consistent. In contrast, when compared with the Kubernetes default scheduler, reinforcement learning-based approaches exhibit consistently higher utilization levels. Among them, EPRS achieves average utilization improvements of 3.57%, 2.42%, and 6.92% for CPU, memory, and disk I/O resources, respectively. In the other two sets of experiments, the performance of the different reinforcement learning-based algorithms in terms of average utilization also differs very little, so we do not provide a comparison of this metric.

Overall, our proposed EPRS improves the load balancing of resources with almost the same performance of average resource utilization as the other algorithms, and improves 25.14% on average in terms of individual resource dimensions compared to D3QN and 31.5% compared to DQN. In terms of the number of Pods, our algorithm is also more balanced, which avoids resource overloading caused by too many Pods on a single node, and system stability is guaranteed.

Our experimental results demonstrate the feasibility and potential research directions of EPRS in edge clustering scenarios. These findings can provide a basis for further load balancing of resources in complex edge clustering environments. Future work could focus on a more balanced and fair distribution of tasks among heterogeneous devices.

## 5. Conclusions and Future Work

In this paper, for the load balancing problem in the dynamic environment of edge clusters, we propose the experience-prioritized reinforcement scheduler (EPRS) and develop an edge cluster scheduling framework that integrates real-time cluster monitoring with reinforcement learning-based decision making. By jointly considering node-level computational resources, memory pressure, storage performance, and container density, EPRS performs online scheduling of Pods without relying on prior knowledge of task execution duration or future workloads.

Through extensive experiments under three representative workload patterns, we observe that EPRS consistently reduces load imbalance across CPU, memory, disk I/O, and container distribution compared with both traditional reinforcement learning baselines and the Kubernetes default scheduler. More importantly, the results indicate that prioritizing historically informative scheduling experiences enables the scheduler to adapt more effectively to dynamic and uncertain task arrivals, leading to more stable resource utilization at the cluster level.

Future research may further investigate how experience-prioritized scheduling behaves under richer workload dynamics, including temporally correlated and bursty task arrivals, and how the learning objective can be extended to jointly reflect load balance, latency sensitivity, and energy efficiency. Examining the generality of experience-prioritized reinforcement scheduling across a broader range of cluster scales and heterogeneous resource configurations may also yield deeper insights into its effectiveness in diverse edge environments.

## Figures and Tables

**Table 1:** Average load imbalance across three experimental workload groups.

Group	Metric	EPRS	D3QN	Dueling DQN	Double DQN	DQN	Kubernetes
Group I	CPU	**9.339**	10.512	10.158	12.893	13.423	9.981
	Memory	7.245	7.455	**6.645**	7.432	7.797	7.838
	Disk	**16.108**	19.713	20.556	25.334	27.143	17.724
	Pods	2	2.625	3.801	3.559	3.496	**1.563**
Group II	CPU	**5.931**	7.323	6.736	8.704	8.666	7.931
	Memory	**5.011**	5.879	6.706	6.941	6.800	9.445
	Disk	**11.845**	14.001	13.069	17.413	17.723	16.192
	Pods	**1.247**	2.108	4.082	3.559	4.216	2.944
Group III	CPU	**5.699**	8.631	8.103	10.069	6.642	9.087
	Memory	**5.394**	8.546	8.177	6.970	6.203	10.388
	Disk	**11.613**	17.315	13.877	18.006	13.331	19.165
	Pods	**1.414**	2.944	3.232	5.617	3.162	3.771

**Bold**: optimal results; Underline: suboptimal results.

## Data Availability

The original contributions presented in this study are included in the article.

## References

[1] Wang Z., Goudarzi M., Gong M., Buyya R. (2024). Deep reinforcement learning-based scheduling for optimizing system load and response time in edge and fog computing environments. Future Gener. Comput. Syst..

[2] Zhang S., He J., Liang W., Li K. (2024). MMDS: A secure and verifiable multimedia data search scheme for cloud-assisted edge computing. Future Gener. Comput. Syst..

[3] Shi W., Cao J., Zhang Q., Li Y., Xu L. (2016). Edge computing: Vision and challenges. IEEE Internet Things J..

[4] García-Valls M., Dubey A., Botti V. (2018). Introducing the new paradigm of social dispersed computing: Applications, technologies and challenges. J. Syst. Archit..

[5] Gong Y., Bian K., Hao F., Sun Y., Wu Y. (2023). Dependent tasks offloading in mobile edge computing: A multi-objective evolutionary optimization strategy. Future Gener. Comput. Syst..

[6] Zhang W., Luo J., Chen L., Liu J. (2023). A trajectory prediction-based and dependency-aware container migration for mobile edge computing. IEEE Trans. Serv. Comput..

[7] Chen Y., He S., Jin X., Wang Z., Wang F., Chen L. (2023). Resource utilization and cost optimization oriented container placement for edge computing in industrial internet. J. Supercomput..

[8] Burns B., Grant B., Oppenheimer D., Brewer E., Wilkes J. (2016). Borg, omega, and kubernetes. Commun. ACM.

[9] Toka L., Dobreff G., Fodor B., Sonkoly B. (2021). Machine learning-based scaling management for kubernetes edge clusters. IEEE Trans. Netw. Serv. Manag..

[10] Kaur K., Garg S., Kaddoum G., Ahmed S.H., Atiquzzaman M. (2019). KEIDS: Kubernetes-based energy and interference driven scheduler for industrial IoT in edge-cloud ecosystem. IEEE Internet Things J..

[11] Ju H., Juan R., Gomez R., Nakamura K., Li G. (2022). Transferring policy of deep reinforcement learning from simulation to reality for robotics. Nat. Mach. Intell..

[12] Li C., Zheng P., Yin Y., Wang B., Wang L. (2023). Deep reinforcement learning in smart manufacturing: A review and prospects. CIRP J. Manuf. Sci. Technol..

[13] Ladosz P., Weng L., Kim M., Oh H. (2022). Exploration in deep reinforcement learning: A survey. Inf. Fusion.

[14] Shakya A.K., Pillai G., Chakrabarty S. (2023). Reinforcement learning algorithms: A brief survey. Expert Syst. Appl..

[15] Sindhu V., Prakash M., Mohan Kumar P. (2022). Energy-efficient task scheduling and resource allocation for improving the performance of a cloud–fog environment. Symmetry.

[16] Rani M., K P S., Jayasingh B.B. (2024). Deep Reinforcement Learning for Dynamic Task Scheduling in Edge-Cloud Environments. Int. J. Electr. Comput. Eng. Syst..

[17] Bandyopadhyay A., Mishra V., Swain S., Chatterjee K., Dey S., Mallik S., Al-Rasheed A., Abbas M., Soufiene B.O. (2024). Edgematch: A smart approach for scheduling iot-edge tasks with multiple criteria using game theory. IEEE Access.

[18] Kristiani E., Yang C.T., Huang C.Y., Wang Y.T., Ko P.C. (2021). The implementation of a cloud-edge computing architecture using OpenStack and Kubernetes for air quality monitoring application. Mob. Netw. Appl..

[19] Goethals T., De Turck F., Volckaert B. (2020). Extending kubernetes clusters to low-resource edge devices using virtual kubelets. IEEE Trans. Cloud Comput..

[20] Parra-Ullauri J.M., Madhukumar H., Nicolaescu A.C., Zhang X., Bravalheri A., Hussain R., Vasilakos X., Nejabati R., Simeonidou D. (2024). kubeFlower: A privacy-preserving framework for Kubernetes-based federated learning in cloud–edge environments. Future Gener. Comput. Syst..

[21] Nguyen Q.M., Phan L.A., Kim T. (2022). Load-balancing of kubernetes-based edge computing infrastructure using resource adaptive proxy. Sensors.

[22] Ali B., Golec M., Murugesan S.S., Wu H., Gill S.S., Cuadrado F., Uhlig S. (2024). GAIKube: Generative AI-based Proactive Kubernetes Container Orchestration Framework for Heterogeneous Edge Computing. IEEE Trans. Cogn. Commun. Netw..

[23] Oleghe O. (2021). Container placement and migration in edge computing: Concept and scheduling models. IEEE Access.

[24] Youn J., Han Y.H. (2022). Intelligent task dispatching and scheduling using a Deep Q-Network in a cluster edge computing system. Sensors.

[25] Li Y., Zhang X., Zeng T., Duan J., Wu C., Wu D., Chen X. (2023). Task placement and resource allocation for edge machine learning: A gnn-based multi-agent reinforcement learning paradigm. IEEE Trans. Parallel Distrib. Syst..

[26] Qiao Y., Shen S., Zhang C., Wang W., Qiu T., Wang X. (2024). EdgeOptimizer: A programmable containerized scheduler of time-critical tasks in Kubernetes-based edge-cloud clusters. Future Gener. Comput. Syst..

[27] Jian Z., Xie X., Fang Y., Jiang Y., Lu Y., Dash A., Li T., Wang G. (2024). DRS: A deep reinforcement learning enhanced Kubernetes scheduler for microservice-based system. Softw. Pract. Exp..

[28] Shen W., Lin W., Wu W., Wu H., Li K. (2025). Reinforcement learning-based task scheduling for heterogeneous computing in end-edge-cloud environment. Clust. Comput..

[29] Cui H., Tang Z., Lou J., Jia W., Zhao W. (2024). Latency-aware container scheduling in edge cluster upgrades: A deep reinforcement learning approach. IEEE Trans. Serv. Comput..

[30] Do H.M., Tran T.P., Yoo M. (2023). Deep reinforcement learning-based task offloading and resource allocation for industrial IoT in MEC federation system. IEEE Access.

[31] Zhang P., Li S., Li D., Ding Q., Shi L. (2025). Sensor-Generated In Situ Data Management for Smart Grids: Dynamic Optimization Driven by Double Deep Q-Network with Prioritized Experience Replay. Appl. Sci..

[32] Lai W.K., Wang Y.C., Wei S.C. (2023). Delay-aware container scheduling in kubernetes. IEEE Internet Things J..

